# Maternal obesity influences Birth Weight more than gestational Diabetes author:

**DOI:** 10.1186/s12884-021-03571-5

**Published:** 2021-02-06

**Authors:** Eman M Alfadhli

**Affiliations:** Medicine department, Taibah University Medical College, PO Box 344, Girls section. Universities Road, T 0966555305170 Medina, Saudi Arabia

**Keywords:** Maternal obesity, Gestational diabetes, Birth weight, Macrosomia, Adverse pregnancy outcomes

## Abstract

**Background:**

Maternal obesity and gestational diabetes (GDM) are commonly encountered during pregnancy. Both conditions are independently associated with unfavorable pregnancy consequences. The objective of this study was to compare the effects of obesity and GDM on birth weight, macrosomia, and other adverse pregnancy outcomes.

**Methods:**

This cohort study involved 531 women with a singleton pregnancy attending the Maternity and Children’s Hospital, Medina, Saudi Arabia, between June 2014 and June 2015. Participants underwent a 75-g oral glucose tolerance test between 24 and 28 weeks. The International Association of Diabetes and Pregnancy Study Groups criteria were used for GDM diagnosis. BMI was assessed at the first antenatal visit, and obesity was defined as a BMI ≥30.0 kg/m2. All women were followed up until delivery. Women were divided into 4 groups: non-GDM nonobese (reference group), GDM nonobese, obese non-GDM, and obese GDM. Clinical characteristics and adverse pregnancy outcomes were compared.

**Results:**

The mean age and BMI of the participants were 30.5 years and 29.3 kg/m2, respectively. GDM was diagnosed in 50.2% of the participants, and obesity was diagnosed in 47.8% of the participants. Obese women with GDM were the oldest and heaviest among all women. The mean birth weight increased in order among the four groups; it was highest in the infants in the obese GDM group, followed by those in the obese non-GDM, GDM nonobese and reference groups. Obesity and GDM alone or in combination were associated with higher rates of macrosomia and cesarean deliveries than the reference group. Neonatal intensive care unit (NICU) admission was higher in infants in the GDM nonobese and obese GDM groups. The frequency of low Apgar score was significantly higher in infants in the obese GDM group than in infants in the reference group.

**Conclusions:**

Maternal obesity seems to influence birth weight more than GDM, while GDM is associated with a greater risk of admission to the NICU. The combination of both conditions is associated with the greatest risk of adverse pregnancy outcomes.

## Background

Maternal obesity and gestational diabetes (GDM) are common metabolic problems in pregnancy. Both conditions are characterized by increased insulin resistance and hyperinsulinemia and are usually diagnosed simultaneously [1].

Maternal obesity has increased considerably among women of reproductive age in the last decades in both high and middle-income countries. In 2014, the estimated percentage of overweight and obesity among pregnant women was 21.7 % in India and 33 % in the United States of America [2]. Likewise, the prevalence of GDM has also increased in parallel to the increase in obesity [3]. The global prevalence of GDM varies widely, from 1–50 %, depending on maternal age, race/ethnicity, socioeconomic status, screening methods, and diagnostic criteria [4]. Applying the International Association of Diabetes and Pregnancy Study Groups (IADPSG) criteria for GDM diagnosis has led to a marked increase in GDM prevalence [5]. The IADPSG recommendations are based on the results of the Hyperglycemia and Adverse Pregnancy Outcome (HAPO) study, which showed a continuous linear association between glucose levels and undesirable pregnancy consequences [6]. As a result, the IADPSG criteria classify GDM with a lower degree of hyperglycemia and treatment of mild GDM has been shown to reduce the frequency of adverse pregnancy outcomes [7, 8]. Although gestational diabetes is routinely screened for and managed throughout pregnancy, obesity is usually overlooked [9]. The lack of intervention that manages obesity during pregnancy, apart from lifestyle modifications, adds to this challenge.

Maternal obesity and GDM are independently linked to unfavorable pregnancy outcomes with some variations in the influence of each condition [10–14]. GDM increases the risk of hypertensive disorders of pregnancy, polyhydramnios, and premature delivery. GDM also causes excessive fetal growth, which increases the risk of cesarean deliveries, shoulder dystocia, and neonatal hypoglycemia. Long-term complications of GDM include diabetes and cardiovascular disease in mothers and obesity and diabetes in the offspring [12]. Maternal obesity increases the risks of gestational diabetes and hypertensive disorders of pregnancy. The fetus is at risk for macrosomia, cesarean deliveries, stillbirth, and congenital anomalies. Long-term complications of maternal obesity include diabetes, hypertension, and cardiovascular disease in mothers and obesity and diabetes in the offspring [2]. Ricart et al. found obesity to affect macrosomia and cesarean delivery more than GDM [14]. In a study from Finland, the risk of macrosomia and cesarean delivery was higher in obese women with and without GDM than in normal-weight women with and without GDM [11]. The HAPO study found that the risk of cesarean delivery was higher in obese women without GDM than in nonobese women with GDM; however, macrosomia was higher in nonobese women with GDM than in obese women without GDM [12].

The purpose of this study was to compare the effects of obesity, GDM and their combination on adverse pregnancy outcomes among Saudi women using the IADPSG criteria. The primary outcomes were birth weight and macrosomia. Secondary outcomes were cesarean delivery, low Apgar score, and neonatal intensive care unit (NICU) admission.

## Methods

A total of 531 pregnant women treated in the antenatal clinic at the Maternity and Children’s Hospital, Medina, Saudi Arabia between June 2014 and June 2015 were included. The inclusion criteria included apparently healthy Saudi women with a singleton pregnancy. Women with pre-existing diabetes or having any chronic diseases that could affect pregnancy outcomes or women who were using any drugs that affect blood sugar were excluded.

The study was approved by the ethics committees of the Maternity and Children’s Hospital, Medina, Saudi Arabia. Written informed consent was obtained from all participants.

At the first antenatal visit, demographic data, height, and weight were collected. BMI was calculated as weight/height squared (kg/m^2^). Obesity was defined as a BMI ≥ 30.0 kg/m^2^ based on the World Health Organization [3]. The mean timing of the first antenatal care visit was 20.63 (8.8) weeks.

Participants underwent a 75-g oral glucose tolerance test (OGTT) between 24 and 28 weeks of gestation. According to the IADPSG recommendations, GDM was diagnosed if any one of the cut-off values were met: fasting plasma glucose 5.1 mmol/L (92 mg/dL), 1-h glucose 10.0 mmol/L (180 mg/dL), or 2-h glucose 8.5 mmol/L (153 mg/dL) [5].

Based on the results of the OGTT and BMI, women were divided into 4 groups: group 1: non-GDM nonobese (normal group); group 2: GDM nonobese; group 3: obese non-GDM; and group 4: obese GDM. Group 1 (non-GDM nonobese) was considered the reference group.

All women were followed up by an obstetrician until delivery. The women who did not have GDM were followed up monthly until the second trimester of pregnancy and then every two weeks during the third trimester. For women with GDM, antenatal visits were occurred every week. In addition, women with GDM were followed by a diabetologist, a diabetes educator, and a dietician. Self-monitoring of blood glucose was performed regularly by women with GDM to ensure adequate glycemic control. Insulin was prescribed when the glycemic target did not achieve. The recommended glycemic targets for fasting and 1-h and 2-h postprandial glucose levels were 5.2 mmol/L (≤ 95 mg/dL), 7.8 mmol/L (≤ 140 mg/dL) and 6.7 mmol/L (≤ 120 mg/dL), respectively [6]. Obese women without GDM were followed as the reference group and not given extra recommendations on diet or exercise.

After delivery, adverse pregnancy outcomes were collected from the medical records.

Comparisons were made between the four groups with regard to clinical characteristics and adverse pregnancy outcomes. The primary outcomes included birth weight and macrosomia. Secondary outcomes were cesarean delivery, low Apgar score, and NICU admission. Macrosomia was defined as a birth weight of 4000 g or more, and an Apgar score of 7 or less at 5 minutes was considered low.

### Statistics

Statistical analyses were performed using SPSS software (v 20.0; SPSS Inc., Chicago, IL). A chi-square analysis was performed to test for differences in the proportions of categorical variables. One-way ANOVA was used to determine the significance of differences between the means of continuous variables. To assess associations of obesity and GDM with pregnancy outcomes, multiple logistic regression was used. The level *P* < 0.05 was taken as the cut-off value for significance.

## Results

**Fig. 1:**
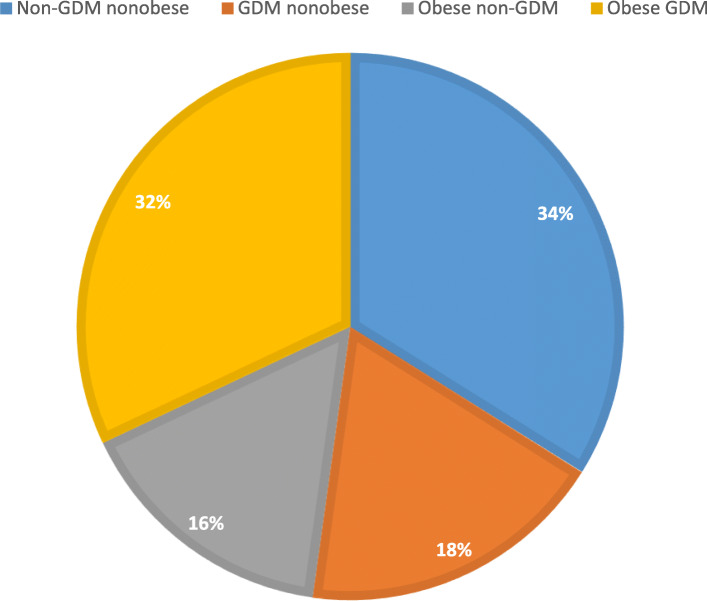
Distribution percentages of the 531 pregnant women among the four groups

The mean age of the women was 30.5 (6.1) years, the mean BMI was 29.3 (6.5) kg/m^2,^ and the mean gestational age at delivery was 38.2 (1.9) weeks. GDM was diagnosed in 50.2 % of the women, of which 63.7 % were obese. Obesity was documented in 47.8 % of all women, of whom 66.7 % had GDM. The four groups’ distribution was as follows: 180 women were non-GDM nonobese, 96 were GDM nonobese, 85 were obese non-GDM, and 170 were obese GDM. Figure 1 shows the distribution percentages of the participants among the four groups.

**Fig. 2:**
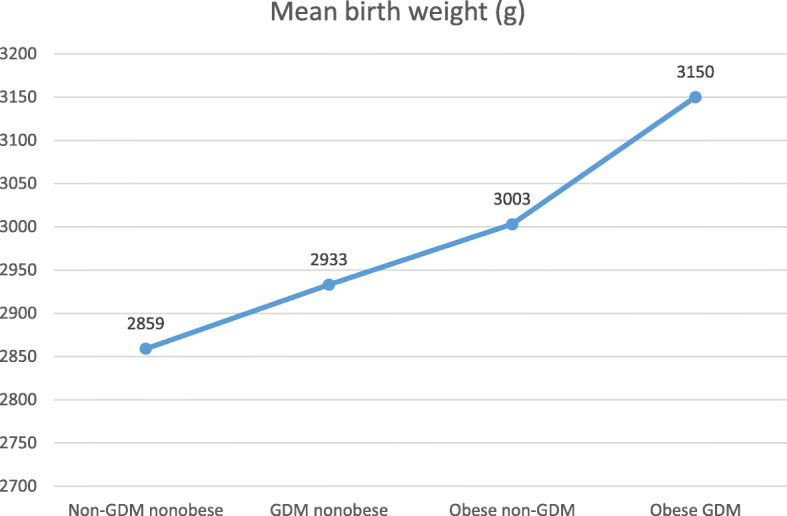
Mean birth weight of the infants in the four groups

Compared to the women in the reference group, women in the other three groups, GDM nonobese, obese non-GDM, and obese GDM, were significantly older and heavier. The mean birth weight increased in order among the groups; it was highest in the infants in the obese GDM group, followed by those in the obese non-GDM, GDM nonobese and reference groups (Fig. 2). However, significance was only reached when the birth weight of infants in the obese GDM group were compared to that of the infants in the reference group, with a 291-gm difference.

The rates of macrosomia and cesarean deliveries were significantly higher in all three groups than in the reference group. Admission to the NICU was higher in all three groups than in the reference group but only reached significance in women with GDM with and without obesity. The frequency of low Apgar score was significantly higher in infants in the obese GDM group than in infants of the reference group (Table 1).

**Table 1:** baseline characteristics and outcomes of the four groups

Variable	*Non-GDM nonobese (*n* = 180)	GDM nonobese (*n* = 96)	Obese non-GDM (*n* = 84)	Obese GDM (*n* = 170)
Age (years) Mean (SD)	28.6 (6.07)	31.7 (6.44)	31.3 (5.78)	33.69 (5.76)
*P* value	-	0.000	0.001	0.000
BMI (kg/m^2^) Mean (SD)	24.2 (3.62)	26.0 (3.01)	34.5 (4.21)	36.2 (5.07)
*P* value	-	0.000	0.000	0.000
Birth weight (gm) Mean (SD)	2859 (0.47)	2933 (0.57)	3003 (0.58)	3150 (0.47)
*P* value	-	0.333	0.062	0.000
Macrosomia Numbers (%)	0 (0)	3 (3.1)	5 (6.0)	7 (4.1)
*P* value	-	0.048	0.013	0.022
Odd ratio (OR)	-	7.22	14.05	9.37
CI	-	0.37-141.52	0.78–252.5	0.5-176.43
Caesarian delivery Numbers (%)	48 (26.7)	42 (43.8)	48 (57.1)	85 (50)
*P* value	-	0.014	0	0
OR	-	2.15	3.69	2.79
CI	-	1.16–3.98	1.97–6.91	1.653–4.722
NICU Admission Numbers (%)	25 (13.9)	29 (30.2)	20 (23.8)	44 (25.9)
P value	-	0.006	0.073	0.016
OR	-	2.68	1.97	2.168
CI	-	1.30–5.52	0.931–4.18	1.143–4.115
Low Apgar Score Numbers (%)	3 (1.7)	5 (5.2)	1 (1.2)	16 (9.4)
*P* value	-	0.171	1	0.006
OR	-	2.82	1	1.858
CI	-	0.56–14.05	0.09–11.2	1.415–2.440

All P values and ORs were compared with the reference group (non-GDM and nonobese women). * reference group. *OR* Odd ratio; *CI* confidence interval. Macrosomia, defined as a birth weight of 4000 g or more. Low Apgar score, defined as an Apgar score of 7 or less at 5 minutes

Table 2 shows comparisons between the three nonreference groups: GDM nonobese, obese non-GDM, and obese GDM. Maternal age was significantly higher in the obese women with GDM than in the women in the GDM nonobese group and the obese non-GDM group, with no differences between the latter two groups. Obese women with GDM were significantly heavier than the women in the other 2 groups. The mean birth weight increased in order from the GDM nonobese, obese non-GDM, and obese GDM groups but only reached significance when comparing the birth weight of infants in the obese GDM group with that of the infants in the nonobese GDM group, with a 217-gm difference. The difference in the mean birth weight of infants between the obese GDM and obese non-GDM groups was not significant. There were no significant differences in the other studied adverse pregnancy outcomes; macrosomia, cesarean delivery, low Apgar score, and NICU admission, between the three groups.

**Table 2:** Comparisons between the three non-reference groups: GDM nonobese; obese non-GDM; and obese GDM

Mean (SD)	Non-GDM Nonobese (group 1) (*n* = 180)	GDM nonobese (group 2) (*n* = 96)	Obese non-GDM (group 3) (*n* = 85)	Obese GDM (group 4) (*n* = 170)	P value		
					Group 2 Vs. Group 3	Group 2 Vs. Group 4	Group 3 Vs. Group 4
Age (year)	28.6 6 (6.07)	31.7 (6.44)	31.3 (5.78)	33.69 (5.76)	0.621	**0.011**	**0.002**
BMI (kg/m^2^)	24.2 (3.62)	26.0 (3.01)	34.5 (4.21)	36.2 (5.07)	**0.000**	**0.000**	**0.009**
Birth weight (mg)	2859 (0.47)	2933 (0.57)	3003 (0.58)	3150 (0.47)	0.488	**0.006**	0.061

## Discussion

In the current study, we found a high prevalence of maternal obesity and GDM among Saudi women: 47.8 % and 50.2 %, respectively. This is consistent with a study from Riyadh in which the prevalence of obesity was 44 % among Saudi pregnant women. In contrast, the prevalence of GDM was 15 % in that study, which is much lower than the rate in this study [13]. The marked difference in GDM prevalence between the two studies is mostly related to the different methods used for GDM diagnosis. While the IADPSG criteria were used in the current study, Wahabi et al. [13] used the Carpenter and Coustan criteria [7]. This finding was demonstrated in our previous study that assessed the prevalence of GDM when applying the IADPSG vs. the Carpenter and Coustan criteria, which revealed a 2.44-fold (144.6 %) increase when applying the IADPSG criteria: 41.5 % vs. 16.9 %, respectively [8]. This is also consistent with the findings from other studies [15, 16].

In the present study, the combination of maternal obesity and GDM affected one-third of women and was associated with older maternal age, higher weight, and more adverse pregnancy outcomes than each condition alone. This is in concordance with many previous studies [10, 13, 14].

The mean birth weight increased in order among the four groups; it was highest in the infants in the obese GDM group, followed by those in the obese non-GDM, GDM nonobese, and reference groups. However, significance was only reached when the infants in the obese GDM group were compared to the infants in the GDM nonobese and reference groups, with 217 and 291 gm differences, respectively. This is consistent with the findings from other studies [10, 11, 13].

The risk of macrosomia and cesarean delivery were significantly increased in all three groups in comparison to that in the reference group. There was a tendency toward a higher risk of macrosomia among infants of obese women with and without GDM than among infants in the nonobese GDM group; however, the result did not reach significance. In the Finnish study, the risk of macrosomia and cesarean delivery were increased in obese women without GDM, and coexistent GDM increased the risk to a greater degree. However, normal-weight women with GDM were similar to normal-weight women without GDM [11]. Similarly, Ricart et al. found that obesity influenced macrosomia and cesarean section rates more than GDM [14]. Although the risk of cesarean delivery was found to be associated more with obesity than GDM in the HAPO study, macrosomia was associated more with GDM than obesity. This contradicting finding is possibly attributed to the lack of medical interventions for mild GDM in HAPO study [12].

The frequency of admission to the NICU was higher in infants in all three groups than in those in the reference group but only reached significance in GDM groups with and without obesity. This is in line with the findings from previous studies [11, 13]. The routine monitoring of infants of GDM mothers due to the concern of neonatal hypoglycemia and close observation of the infants’ blood sugar may contribute to the increased risk of NICU admission. Nonetheless, in the Finnish study, the risk of NICU admission remained elevated in the infants of mothers with GDM after adjustment for neonatal hypoglycemia [11].

The frequency of low Apgar score was significantly higher in the infants of obese GDM women than in the infants of the reference group. In addition, there was a tendency toward a low Apgar score in the GDM nonobese group in comparison to the scores in the obese non-GDM group and the reference groups; however, the findings did not reach significance. This result is consistent with the findings of the Wahabi et al. and Finnish studies [11, 13]. Although Hildeń et al. found that maternal obesity and GDM are major independent risk factors for a low Apgar score, no interaction effect between GDM and obesity was found [10].

From the findings of the current study and others, one can extrapolate that obesity is associated with a higher birth weight and greater risk of macrosomia and cesarean delivery than GDM. On the other hand, GDM is associated with a greater risk of low Apgar score and admission to the NICU. However, the combination of obesity and GDM is associated with the greatest risk of adverse pregnancy outcomes [10, 11, 13].

Although obesity is recognized to adversely affect pregnancy, obesity during pregnancy is usually overlooked. Maternal obesity should indicate a high-risk pregnancy, particularly if combined with GDM. Lifestyle interventions, including diet and physical activity, should be recommended for obese women during pregnancy. Weight monitoring during pregnancy is required to avoid excessive weight gain. Women of reproductive age with obesity should receive facts and advice about the risks of obesity during pregnancy and be recommended to lose weight before and between pregnancies [17].

Limitations of the present study include the lack of data on prepregnancy maternal weight, which might be a contributing factor to the higher frequency of obesity in this cohort of participants. A second limitation is that we did not look at the gestational weight gain which could influence the pregnancy outcomes. Another limitation was that no intervention was provided to the obese non-GDM group, which may contribute to the higher frequency of increased birth weight of the infants in this group. The strength of this study is that we followed the participants prospectively, so we had the chance to ensure the maintenance of reasonable glycemic control among women with GDM. In addition, an OGTT was performed on all participants, so no woman with GDM was missed.

## Conclusions

Maternal obesity may be associated with a higher birth weight and a greater risk of macrosomia and cesarean delivery than GDM. Conversely, GDM may be associated with a greater risk of low Apgar score and admission to the NICU. However, the combination of obesity and GDM is associated with the greatest risk for all adverse outcomes. Further studies are needed to confirm our results.

Maternal obesity should indicate a high-risk pregnancy, particularly if combined with GDM. To reduce the risk of adverse pregnancy outcomes, weight monitoring and lifestyle modification, including diet and physical activity, should be recommended for obese pregnant women. Future works are needed to study the effect of such modifications on pregnancy outcomes.

## Data Availability

Datasets obtained and/or analyzed in this study are available from the corresponding author on reasonable request.

## Abbreviations

GDM: Gestational diabetes
OGTT: Oral glucose tolerance test
IADPSG: International Association of Diabetes and Pregnancy Study Groups
NICU: Neonatal intensive care unit
